# Cervical cancer control and prevention in Malawi: need for policy improvement

**DOI:** 10.11604/pamj.2015.22.247.6439

**Published:** 2015-11-17

**Authors:** Fresier Chidyaonga Maseko, Maureen Leah Chirwa, Adamson Sinjani Muula

**Affiliations:** 1 School of Public Health and Family Medicine, Department of Community Health, University of Malawi, College of Medicine, Mahatma Gandhi Road, Private Bag 360, Chichiri, Blantyre 3, Malawi; 2 Prime Health Consulting and Services, Prime Health Consulting and Services A47/5/240, Malingunde Road, Lilongwe, Malawi

**Keywords:** Cervical cancer, policy, cervical cancer control and prevention

## Abstract

**Introduction:**

Malawi has the highest incidents of cervical cancer followed by Mozambique and Comoros thus according to the 2014 Africa cervical cancer multi indicator incidence and mortality score card. Despite having an established cervical cancer prevention program, there is low screening coverage. Studies have been carried out to determine socio-cultural and economical barriers to cervical cancer prevention services utilization and very few have concentrated on health system and policy related barriers to cervical cancer prevention and control. The paper presents finding on a qualitative study which carried out to determine the suitability of the national sexual and reproductive health and rights [SRHR] in mitigating challenges in cervical cancer control and prevention.

**Methods:**

a desk review of the Malawi National Sexual and Reproductive Health and Rights [SRHR] policy 2009 was done with an aim of understanding its context, goal and objectives. Analysis of the policy history provided insight into the conditions that led to the policy. Policies from countries within the region were referred in the review. Government officials were interviewed to solicit information on the policy.

**Results:**

Malawi does not have a standalone policy on cervical cancer; however, cervical cancer is covered under reproductive cancer theme in the SRHR. Unlike some policies within the region, the Malawian SRHR policy does not mention the age at which the women should be screened, the frequency and who is to do the screening. The policy does not stipulate policy implications on the ministry of health, the SRH programs and health service providers on cervical cancer. Furthermore the policy does not include HPV vaccination as a key component of cervical cancer control and prevention.

**Conclusion:**

the policy does not reflect fairly the best attempt to reduce the incidence and mortality of cervical cancer as such we recommend that the Reproductive Health Directorate to consider developing a standalone policy on cervical cancer control and prevention.

## Introduction

Globally, cervical cancer is the fourth most common cancer in women. Statistics shows that 528,000 new cases were diagnosed worldwide in 2012 and Malawi had the highest rate of cervical cancer, followed by Mozambique and Comoros [1]. According to the 2014 Africa cervical cancer multi indicator incidence and mortality score card, Malawi has an incidence of 75.9 per 100000 women and a mortality rate of 49.8 per 100000 across 10 age groups [2].The incidence is highest among women aged around 40 years [3]. Statistics show that 3684 women are diagnosed with cervical cancer annually in Malawi and 2314 die from the disease [4]. Regardless of these alarming statistics, the country has had an established cervical cancer prevention program which has been running since the late 1980s [3, 5]. In accordance with the World Health Organization's (WHO's) recommendation for cervical cancer control in resource-poor countries and through the Ministry of Health-Reproductive Health Directorate (MoH-RHD) and its partners, Malawi adopted a cost-effective strategy for cervical cancer prevention and control. The ministry introduced cervical cancer screening program using Visual Inspection with Acetic acid (VIA) in 2004 after being piloted between 1999 and 2001 in two districts, one rural and the other urban [6–8]. Since then, the program has been scaled up to all districts and central hospitals [3]. According to MoH-RHD statistics of June 2014, over 100 health facilities across the country were providing VIA services [9]. Cumulatively, a total of 59,217 women have been screened of which 5,744 were VIA positive representing 9.7percent.However, 1,777 representing 2.9% had suspected cervical cancer [10]. From these statistics, and considering the number of women at risk of developing cervical cancer in Malawi, on the other hand taking into account that the cervical cancer prevention and control program has been in existence for over two decades, one might not be wrong to conclude that the program has been experiencing significant challenges such that it is not able to address satisfactorily the existing cervical cancer challenges in the country. Many of the studies that have been conducted to determine the coverage of cervical cancer in Malawi have focused on socio-cultural and economical barriers to cervical cancer prevention services utilization [3, 7, 11] and very few have concentrated on health system and policy related barriers to cervical cancer prevention and control [12]. This paper presents finding on a qualitative study which was carried out to determine the suitability of the national sexual and reproductive health and rights (SRHR) in mitigating challenges in cervical cancer control and prevention in Malawi.

## Methods

A desk review of the National Sexual and Reproductive Health and Rights (SRHR) policy 2009 was done in regards to cervical cancer with an aim of understanding its context, goal and objectives. Analysis of the policy history was done to provide insight into the conditions that led to the policy. The review focused much on the following attributes: At what age does the policy state that women should begin screening for cervical cancer? How often does the policy state that women should be screened? The type of cervical cancer screening to be used; The cadre of health workers to provide the cervical cancer services. Comparison, with regards to cervical cancer, was also made with similar policies from countries within the region. Compared were the National Policy for Reproductive Health (NPRH) of Namibia and the SRHR policies from the Kingdom of Swaziland, Zambia and the cervical cancer policy of the republic of South Africa. Key informant interviews were also conducted with ministry officials to solicit information on whether the ministry or any government agency ever reviewed, evaluated or proposed revision for the SRHR to assure that it reflects current scientific knowledge and best practices for achieving compliance. We also asked them if at all the ministry of health engaged national, local, and special interest advocacy groups when developing policy. Information on who participated (i.e. persons or representatives of stakeholder groups) in the setting the SRHR policy agenda or in defining and prioritizing health needs and services at the national and local levels was also solicited. The ministry officials were from the RHD and the office responsible for non-communicable diseases at the ministry headquarters. A key informant interview guide was used in interviewing the officials.

## Results

According to the policy document, the goal of the SRHR policy is to provide a framework for provision of accessible, acceptable and affordable, comprehensive SRHR services to all women, men and young people of Malawi through informed choice to enable them attain their reproductive rights and goals safely. The SRHR policy (2009) originated from the Reproductive Health (RH) policy which the MoH-RHD developed in 2002. The revision of the RH policy into SRHR policy was based on the Maputo Plan of Action [13]. Like many other SRHR policies in the Southern Africa Developing Community (SADC) region, the policy was initiated to accommodate several components of SRHR which were not included in the RH policy. These components included the Basic Emergency Obstetric and Neonatal Care (BEmONC); Community Based Maternal and Neonatal Care; Cervical Cancer Screening; Youth Friendly Health Services, Anti-Retroviral Therapy, and Prevention of Mother to Child Transmission (PMTCT) [14]. Like the SRHR policy of the kingdom of Swaziland, in which the policy statement covering cervical cancer falls under the policy theme of “cancer of the reproductive system”, in the Malawi SRHR policy, cervical cancer is covered under “reproductive cancer policy” theme [15, 16]. The reproductive cancer policy theme aims at reducing the incidence and complications of cancers of reproductive organs in all men and women. Unlike the South African cervical cancer policy, the policy statements on cervical cancer in the Malawi SRHR policy document do not mention about the age at which women should begin screening and neither mention on how often the women should come for screening. Furthermore, the policy does not mention what type of screening test should be used and which cadre of health professionals should be directly involved in providing screening and treatment services. However, like the South African policy, the Malawi national SRHR policy does mention that screening for cervical cancer shall be integrated in primary health care and routinely offered to all women at all levels of health care [17, 18]. Like the Namibian National Policy for Reproductive health, the Zambian and the Swazi's National SRHR policies, the Malawi SRHR policy does not include HPV vaccination. It was also noted that unlike other SRHR policies within the region, the Malawian policy does not stipulate the policy implications on the ministry of health, the SRH programs and health service providers on cervical cancer [15]. Information obtained from the interview with a ministry official indicated that all the necessary steps needed to develop a policy were followed when this policy was developed. When the need to revise the RH policy was identified, the MoH-RHD was given the mandate to lead in the policy development. It then constituted a committee which was given the responsibility of leading the policy review and development. The committee was composed of experts from different institutions such as university of Malawi, international and local non-governmental organizations, government departments and the MoH-RHD staff. One of the responsibilities of the committee was to design the policy which involved identification of the guiding principles of the policy, a definition of vision, and clear aims and objectives. The team gathered the necessary information needed for the drafting of the policy and described an overall strategic approach to the policy. The interviewee also reported that the committee conducted both internal (within the ministry of health) and external consultations in order to solicit information from stake holders. Review of the SRHR policy (2009) is scheduled for 2015.

When asked to comment on the SRHR policy in relation to cervical cancer, a non-communicable diseases (NCDs) unit official at the MoH headquarters said.

*“…Yes indeed we have the SRHR policy which covers cervical cancer; yes we can say that we have a policy which is covering cervical cancer. You know a policy is supposed to be broad. You can find the details on cervical cancer in the national cervical cancer strategy. Unfortunately, our strategy document does not holistically address the issues of cervical cancer in Malawi. The strategy, as is the case with the policy, only provides information on the screening. The two documents do not tackle other critical areas on cervical cancer management which include palliative care and vulnerable groups, such as women living with HIV. The documents really needed to address issues on primary prevention which should include how awareness will be raised and information disseminated”*.

Asked on the same, an official from the MoH-RHD, who also participated in the development of the SRHR policy document, commented that the SRHR policy on its own cannot be seen to holistically address the challenges cervical cancer control and prevention program is facing in the country, however the SRHR policy document is supported by other documents such as the National Service Delivery Guidelines for Cervical Cancer Prevention of 2005 which stipulate the strategies which have to be put in place to combat cervical cancer in Malawi. These documents also stipulate at what age a woman can be screened, where the women can go for the screening, what screening test they will get and who is qualified to do the screening.

*“…YSo the policy was just a guiding document we could not put all those specifics in there but otherwise aah (clearing the throat) we would say that it is recommended to start screening at age 35, but as a country we have actually seen that there are cases being reported at 20s. So if somebody at the age of 20 walks into a facility there's no health worker who is going to send them back to say why have you come for screening” We are starting at such, such an age”…” All ministry officials admitted that although there are other policy documents such as the National Service Delivery Guidelines for Cervical Cancer Prevention* [19] *that complements the SRHR policy in mitigating cervical cancer challenges in Malawi, still there are policy deficiencies in some areas of cervical cancer prevention and control*.

## Discussion

Cervical cancer prevention and control is made up of several key components ranging from community education, social mobilization, vaccination, screening, and treatment to palliative care [20]. However our review of the policy has shown that the Malawi SRHR policy, unlike other SRHR policies within the region, does not address all important components of cervical cancer prevention and control and as such does not fully address challenges in cervical cancer control and prevention in prevailing in the country. The policy does not reflect fairly the best attempt to reduce the incidence of cervical cancer and the morbidity and mortality associated with it. Our findings show that the policy lacks information on the type of screening test(s) which should be used and the cadre of health professionals to administer the screening. Furthermore the policy does not recommend the age at which women should start screening. At this time when Malawi is piloting HPV vaccine as one preventive measure against cervical cancer, the policy does not mention anything about the vaccine. This might be understandable considering the fact that at the time the policy was developed, the country had not yet started offering HPV vaccines to the girls. On the other hand cervical cancer screening was never regarded as a priority up until 2012 when it was included in the essential health package under the 2012-16 Health sector strategic plan [21]. However this calls for inclusion of HPV vaccine in the future policy. Findings from this study show that in Malawi, women below the age of twenty years were able to access cervical cancer screening services in public health facilities as long as they demanded the services. Much as this can be considered as a good practice to detect pre-cancerous abnormalities at an early stage, a substantial body of evidence has shown that screening in women younger than 25 years of age has little or no impact on the risk of developing invasive cancer [22]. This practice is not cost effective to a country like Malawi with scares and limited resources more especially in this post-HPV vaccination era.

## Conclusion

In view of the findings and considering the increasing incidence of cervical cancer and the high mortality rates of cervical cancer patients in Malawi, it is our recommendation that the MoH-RHD should consider developing a standalone comprehensive national policy on cervical cancer management. The policy should include all the components of cervical cancer control and prevention. At minimum, the policy should tackle issues on primary prevention, including determining how awareness will be raised and information disseminated, and providing guidance on HPV vaccines. The policy should also cover secondary prevention or screening, including the age at which careening should start and the frequency of the screening. Diagnosis, treatment protocols for pre-cancerous lesions and invasive cervical cancer, provision of palliative care and the needs of particularly vulnerable groups, such as those living with HIV should also be covered in the policy [23]. Developing such a policy would signal government's commitment to addressing cervical cancer in the country, identify the steps that need to be taken, and indicate the funding and resources needed to address cervical cancer. The policy will guide and unify various stake holder efforts in the struggle against an increasing cervical cancer burden. On the other hand the policy will also save as a legal framework for service users, NGOs and activists to base their arguments on, when advocating for more financial and technical support from government and its partners that are playing different roles in combating cervical cancer in Malawi.
